# Dosing Regimen for Cefotaxime Should Be Adapted to the Stage of Renal Dysfunction in Critically Ill Adult Patients—A Retrospective Study

**DOI:** 10.3390/antibiotics13040313

**Published:** 2024-03-29

**Authors:** Théo Dillies, Sophie Perinel-Ragey, Patricia Correia, Jérôme Morel, Guillaume Thiery, Manon Launay

**Affiliations:** 1 Service de Médecine Intensive et Réanimation G, Centre Hospitalier Universitaire (CHU) deSaint-Etienne, F-42055 Saint Etienne, France; 2 SAINBIOSE U1059, Université Jean Monnet, INSERM, F-42023 Saint Etienne, France; 3 Service de Réanimation Polyvalente B, CHU de Saint-Etienne, F-42055 Saint Etienne, France; 4 Research on Healthcare Performance RESHAPE, INSERM U1290, Université Claude Bernard Lyon, F-69008 Lyon, France; 5 Centre Régional de Pharmacovigilance, CHU de Saint-Etienne, F-42055 Saint Etienne, France

**Keywords:** cefotaxime, renal function, therapeutic drug monitoring, augmented renal clearance, personalised medicine

## Abstract

Cefotaxime administration is recommended in doses of 3–12 g/day in adults with a Glomerular Filtration Rate (GFR) > 5 mL/min. This study aimed to assess the impact of renal function and obesity on cefotaxime concentrations in intensive care unit (ICU) patients. A retrospective cohort study was conducted on consecutive ICU patients receiving continuous cefotaxime infusion between 2020 and 2022 [IRBN992021/CHUSTE]. Doses were not constant; consequently, a concentration-to-dose ratio (C/D) was considered. Statistical analysis was performed to assess the relationship between cefotaxime concentrations, renal function, and obesity. A total of 70 patients, median age 61 years, were included, with no significant difference in cefotaxime concentrations between obese and non-obese patients. However, concentrations varied significantly by GFR, with underdosing prevalent in patients with normal to increased renal function and overdosing in those with severely impaired renal function. Adjustment of cefotaxime dosing according to GFR was associated with improved target attainment. Cefotaxime dosing in critically ill patients should consider renal function, with higher initial doses required in patients with normal to increased GFR and lower doses in those with severely impaired renal function. Therapeutic drug monitoring may aid in optimising dosing regimens. Prospective studies are warranted to validate these findings and inform clinical practice.

## 1. Introduction

Cefotaxime is a broad-spectrum beta-lactam antibiotic that is usually recommended for the empirical treatment of critically ill patients [1,2]. A target concentration of free beta-lactam in plasma between four and eight times the minimum inhibitory concentration (MIC) of the causative bacteria for 100% of the dosing interval (fT ≥ 4–8 × MIC = 100%) has been proposed to maximise bacteriological and clinical response in intensive care unit (ICU) patients. For undocumented infections, the target cefotaxime blood concentration range is therefore between 25 and 60 mg/L [3].

Cefotaxime is partially metabolised to desacetyl-cefotaxime prior to excretion. The majority of a cefotaxime dose is excreted in the urine—approximately 60% as an unchanged active substance, and a further 24% as desacetyl-cefotaxime. However, according to the Summary of Product Characteristics (SmPC), cefotaxime should be administered in all adults with a glomerular filtration rate (GFR) of more than 5 mL/min/1.73 m^2^ at a dose of 3 g/day up to 12 g/day, depending on the severity and location of the infection.

However, sepsis favours acute renal failure, which occurs in 40% of critically ill patients and reduces renal clearance of antibiotics [4]. Conversely, septic shock in its initial phase and treatments such as resuscitation fluids and positive inotropic agents contribute to an increase in renal blood flow and, consequently, to an augmented renal clearance (ARC) in ICU patients (defined by GFR > 130 mL/min/1.73 m^2^) [3]. ARC has been found in 30–40% of ICU patients, and even more frequently in patients in neurocritical ICUs and trauma units [5]. Since beta-lactams are hydrophilic antibiotics mainly excreted via the kidneys, an increase in their renal clearance generally leads to lower plasma concentrations [3]. Conversely, beta-lactam clearance may be significantly reduced in acute kidney injury [3]. The first aim of this study was to investigate the impact of such changes in renal function on early cefotaxime concentrations in the ICU.

In addition, the prevalence of obesity has tripled worldwide and continues to increase [6]. Obesity is defined as a body mass index (BMI) of more than 30 kg/m^2^. In ICU, the proportion of obese patients is now 20% [7,8]. Obese ICU patients have a different prognosis than non-obese patients, and exposure to certain beta-lactams has been shown to be different in obese and non-obese patients [3,9]. For example, we have recently shown that ceftazidime concentrations were significantly lower in obese patients at similar doses [10]. Weight also affects the calculation of body surface area, and the GFR estimated by the CKD-EPI (Chronic Kidney Disease—Epidemiology Collaboration) formula is normalised to a body surface area of 1.73 m^2^. Weight should consequently have an influence on the assessment of GFR according to the CKD-EPI formula. Our second aim was, therefore, to investigate the independent effect of obesity on cefotaxime concentrations.

## 2. Results

### 2.1. Basic Characteristics

A total of 70 patients (49 men, 34 obese, median age 61 years) were included. The most common site of bacterial infection was the lung (51/70), and 26 patients (37%) had SARS-CoV-2 infection. Baseline data and identified pathogens are shown in Table 1. The median length of ICU stay was 19 (11–31) days. The median SOFA score before the introduction of cefotaxime was 9 (5–11). Overall, 15 patients (21%) died before leaving the ICU.

### 2.2. No Impact of Obesity on Cefotaxime Concentration

Steady-state cefotaxime concentrations at first blood draw did not differ significantly by obesity status (median concentrations of 31.5 mg/L in obese vs. 34.0 mg/L in non-obese patients, *p* = 0.7599), with similar doses (8000 and 6000 mg/d in obese and non-obese patients, respectively, *p* = 0.4791) and GFR (95.5 and 105.5 mL/min/1.73 m^2^ in obese and non-obese patients, respectively, *p* = 0.0940). Concentrations were monitored at similar times in obese and non-obese patients (38.4 h vs. 40.5 h, *p* = 0.3532).

### 2.3. Significant Impact of Renal Function on Cefotaxime Concentration

**Figure 1:**
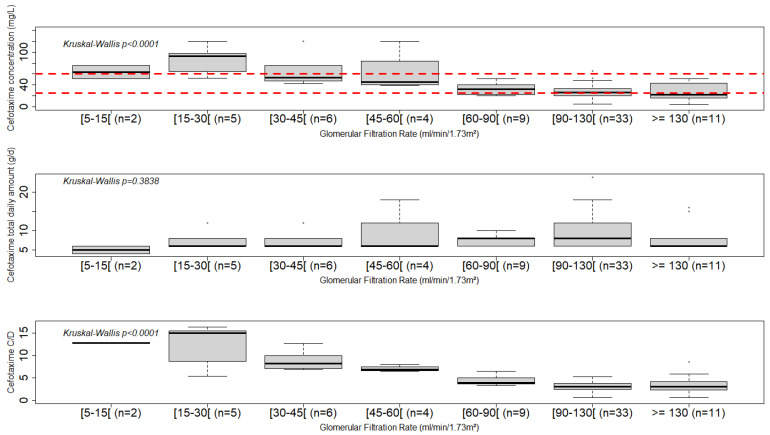
First concentration of cefotaxime (mg/L), cefotaxime daily dose (mg/d) and dose-adjusted concentration ratios of cefotaxime as a function of GFR (mL/min·1.73 m^2^). The dashed lines represent cefotaxime target range for undocumented infections (25–60 mg/L).

Figure 1 shows the initial concentrations, total daily doses and C/D ratios of cefotaxime as a function of GFR. Cefotaxime concentrations were significantly different according to GFR (Kruskal–Wallis test, *p* < 0.0001), with 64 ± 17 mg/L (median = 64 mg/L) at GFR between 5 and 15 mL/min/1.73 m^2^ to 27 ± 16 mg/L (median = 22 mg/L) at GFR above 130 mL/min/1.73 m^2^. The time between the administration of cefotaxime and its monitoring did not differ significantly by GFR level (*p* = 0.8775), with a median delay of 38.5 h after initiation.

**Figure 2:**
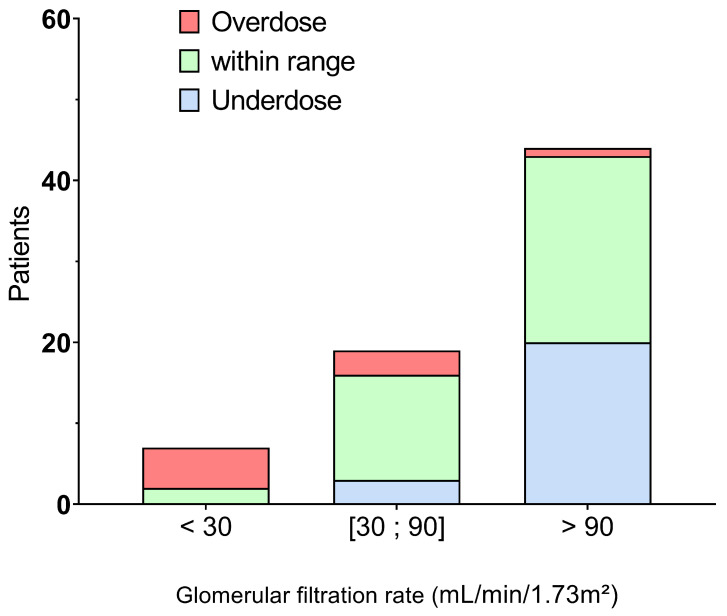
Achievement of the target range for patients with a GFR of less than 30 mL/min·1.73 m^2^, between 30 and 90 mL/min·1.73 m^2^ and more than 90 mL/min·1.73 m^2^. Overdosed patients were drawn in red, underdosed patients in blue, and patients within range were drawn in green.

**Figure 3:**
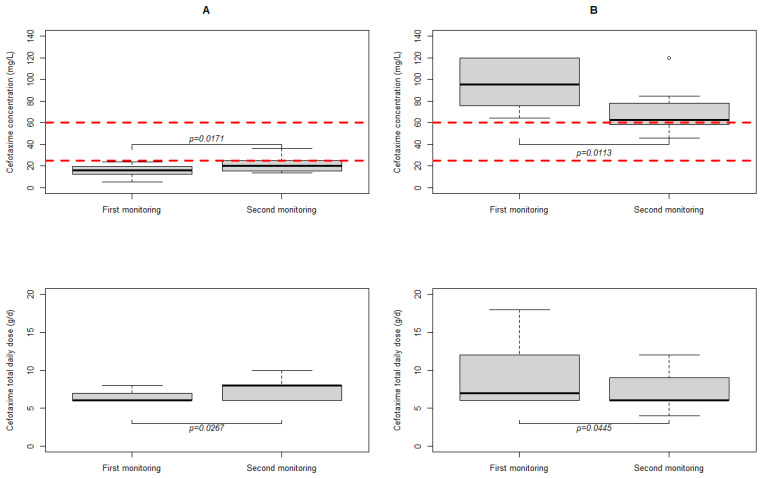
Comparison of cefotaxime concentrations and doses between the first and second monitoring, for patients with underdosing at the first monitoring ((**A**), *n =* 12 patients with GFR between 88 and 146) and patients with overdose at first monitoring ((**B**), *n =* 8 with a GFR between 12 and 46). The dashed lines are the target range for cefotaxime in undocumented infections (25–60 mg/L). The median delay between first and second monitoring was 47.5 h.

Cefotaxime concentrations were within the usual target range for only 54% of the patients (38/70). Cefotaxime underdose and overdose were detected in 33% (23/70) and 13% (9/70) of patients, respectively. As shown in Figure 2, underdosing was most common in patients with a GFR above 90: 45% (20/44) at first cefotaxime monitoring. The median calculated C/D value was 3 for patients with GFR above 90. In other words, a minimum dose of 8 g would be required to achieve a concentration in the target range (25–60 mg/L). A total of 12 of these 23 underdosed patients were monitored a second time (see Figure 3A), with GFR ranging from 88 to 146 mL/min/1.73 m^2^. Doses were significantly higher at the second sampling time point (8 g versus 6 g, *p* = 0.0267), resulting in higher cefotaxime concentrations (20.0 mg/L versus 16.1 mg/L, *p* = 0.0171). The C/D ratio was therefore similar (3 vs. 3, *p* = 0.7334). However, only 3/12 (25%) of patients reached the target range.

Overdose occurred most frequently in patients with a GFR of less than 30, with five of the seven patients with a GFR < 30 experiencing an overdose. The median C/D ratio was 13. To achieve a concentration in the target range, patients with a GFR < 30 would need theoretical total daily doses of 2 to 4.5 g. A total of eight of these nine overdose patients were monitored a second time (see Figure 3B), with GFRs between 12 and 46 mL/min/1.73 m^2^. The dose was significantly lower at the second measurement (6 g versus 7 g, *p* = 0.0445), resulting in lower cefotaxime concentrations (62.4 mg/L versus 95.4 mg/L, *p* = 0.0113). The C/D ratio was similar (11 vs. 13, *p* = 0.3828). However, only three out of eight (37.5%) patients reached the target range.

Patients with a GFR between 30 and 90 were mostly in the target range (3/19 with underdosing and 3/19 with overdosing). The median C/D ratio was 6, which explains that the target range was mostly successfully reached with the standard dose of 6 g daily.

## 3. Discussion

In this study, only 54% of the ICU patients reached the target concentration at the first measurement with cefotaxime standard regimen. Although cefotaxime is widely used in intensive care units, no adjustment for renal function is currently made. As described in the prescribing information, no significant difference in total daily dose as a function of GFR is considered. However, in this study, a significant correlation between GFR and cefotaxime concentration was found in 70 ICU patients, which is supported by recent studies showing that GFR is a significant covariate influencing cefotaxime clearance [11,12] and poor target attainment [13]. As a result, patients with severely impaired renal function were mostly overdosed (71%, 5/7 patients) and patients with normal to increased renal function were frequently underdosed (45%, 20/44).

ARC was observed in 20% of patients. ARC is indeed a common phenomenon in critically ill patients. Drug excretion is increased in these patients, especially for hydrophilic antibiotics that are mainly excreted via the kidneys, such as beta-lactams. ARC has already been described as a risk factor of beta-lactam underdosing [14]. Without an initial dose adjustment to renal function, appropriate antibiotic therapy is delayed. Increased doses have been already proposed for another third generation cephalosporin (i.e., ceftriaxone) in patients with ARC [13,15,16]. For cefotaxime, our results suggest that 8 g should be considered as a minimum initial daily dose in critically ill patients with normal to increased renal function. This suggestion is supported by data on critically ill children where 200 mg/kg using continuous infusion was associated with better probability of target attainment in ARC children with a weight of 40 kg or more [17]. However, only 25% of previously underdosed patients reached the target range at the second cefotaxime monitoring with 8 g, implying that higher doses could also be considered. As delaying the first appropriate antibiotic administration has been associated with increased in-hospital mortality [18], this strategy should improve optimal treatment of infections and, therefore, length of hospital stay and treatment outcomes [19].

Considering that cefotaxime concentrations were too high in 71% of patients with severely impaired renal function, lower doses, e.g., 2–4.5 g, should be administered. Although the dose was reduced slightly from 7 g to 6 g at the second measurement, only 37.5% of patients reached the target range and the overdose usually persisted. As the patients were mostly sedated, overdose-related neurotoxicity was not investigated.

The SmPC of cefotaxime and studies in critically ill children suggest that weight-normalised doses for cefotaxime should be considered [13,20]. However, cefotaxime concentrations did not differ significantly between our obese and non-obese critically ill adult patients who used similar doses and had similar GFRs. Therefore, weight-normalised doses should be avoided in adults.

Several limitations should be noted. First, this is a retrospective study of a small population. TDM was routinely performed at steady state, without a program to ensure regularity, especially 24–48 h after the start of cefotaxime treatment and when significant changes in renal function occurred. As is currently standard practice in most hospital laboratories, desacetylcefotaxime, the main metabolite of cefotaxime, and the unbound fraction of cefotaxime were not monitored. In addition, renal function was not assessed as recommended in the internationally recognised KDIGO (Kidney Disease Improving Global Outcomes) recommendations.

## 4. Patients and Methods

### 4.1. Patients

This retrospective cohort study was conducted between December 2020 and March 2022 at Saint Etienne University Hospital (France). The study was approved by the Institutional Review Board [IRBN992021/CHUSTE], and all patients received an information letter. All consecutive critically ill patients with continuous cefotaxime infusion monitored at steady state (target range: 25–60 mg/L) were enrolled in this study.

### 4.2. Cefotaxime Treatment and Monitoring

The initial dose administered to patients was left to the physician’s initiative and was approximately 100 mg/kg/day (200–300 mg/kg/day for neuromeningeal infections). Therapeutic drug monitoring (TDM) was performed routinely and at the physician’s discretion. Doses may have been adjusted according to the physician’s decision, justifying consideration of the dose-adjusted concentration ratio (C/D) to compare cefotaxime exposure in patients receiving different doses. The C/D ratio of cefotaxime was calculated by dividing the blood concentration of cefotaxime (expressed in milligrammes per litre) by the total daily dose (in grammes). Based on the median C/D ratio and linear pharmacokinetics, the theoretical dose to achieve 25 to 60 mg/L was calculated using the following formula:

$$C/D\;ratio=\frac{Concentration}{Dose}↔Dose=\frac{Concentration}{C/D\;ratio}$$

### 4.3. Collected Data

Demographic data, cefotaxime treatment regimen, clinical and laboratory results were collected retrospectively from medical records. Laboratory tests included albumin, total protein, C-reactive protein, creatinine concentration, and GFR estimated using the CKD-EPI formula. Disease severity was defined using the Sequential Organ Failure Assessment (SOFA) score. Obesity was defined as a BMI of 30 kg/m^2^ or more. 

### 4.4. Statistical Analysis

Statistical analysis was performed using the program R (R Foundation for statistical computing, version 3.2.3, Vienna, Austria). For continuous variables, deviation from normality was tested using the Shapiro goodness-of-fit test, and variables were described as mean (or geometric mean) ± standard deviation (together with median, interquartile range and extreme values). As the distribution of cefotaxime concentrations deviated significantly from normality, non-parametric tests were applied: Mann–Whitney test for two-group comparisons (male vs. female, obese vs. non-obese) and Kruskal–Wallis test to compare cefotaxime concentrations by renal function levels. Data from patients with two consecutive monitoring periods were compared using the Wilcoxon Signed-Rank Test.

## 5. Conclusions

Depending on GFR, the concentration of cefotaxime at standard doses varies by a factor of 1 to 3. Consequently, more than 36 h after antibiotic initiation, only 54% of critically ill patients reach the target concentration. GFR was significantly related to cefotaxime exposure, even in ARC patients, and clinicians should be aware of this point as cefotaxime is often prescribed without renal adjustment for GFR > 5 mL/min, as recommended in the prescribing information. The cefotaxime dosing strategy of a reduced dose of 2–4.5 g for severely impaired GFR and a dose of 8 g for ARC should be considered. In these cases, TDM may be essential. As these data are observational and retrospective, prospective clinical studies are needed to confirm these critical findings.

## Figures and Tables

**Table 1:** Population characteristics. All data shown were collected at the time of cefotaxime monitoring. BMI: Body mass index, eGFR: estimated glomerular filtration rate, CKD-EPI: Chronic Kidney Disease Epidemiology Collaboration, MV: mechanical ventilation, RRT: renal replacement therapy, ECMO: extracorporeal membrane oxygenation.

Characteristics	Values Median (IQR) [Range] or Number (%)
Age (years)	61 (50–70) [18–77]
Gender (M)	49 (70%)
BMI (kg/m^2^)	29.8 (25.6–36.5) [17.8–61.6]
<30	36 (51%)
≥30	34 (49%)
Blood creatinine (μmol/L)	63.5 (45–94) [27–445]
eGFR using CKD-EPI formula (mL/min/1.73 m^2^)	98 (63–113) [8–152]
<30	7 (10%)
[30–60[	10 (14%)
[60–90[	9 (13%)
[90–120[	30 (43%)
≥120	14 (20%)
Blood proteines (g/L)	61 (56–66) [41–75]
Reason for admission	
Medical	26 (37%)
COVID	26 (37%)
Surgical	18 (26%)
Site of infection	
Pulmonary	51 (72%)
Neuromeningeal	10 (14%)
Urinary	4 (6%)
Osteoarticular/soft tissues	1 (2%)
Blood cultures	2 (3%)
Abdominal	2 (3%)
Types of germs identified (60/70 patients)	84
Gram positive cocci	22 (26%)
*Staphylococcus* spp.	8 (9%)
*Streptococcus* spp.	13 (16%)
*Enterococcus* spp.	1 (1%)
Gram negative cocci	2 (2%)
*Branhamella* spp.	2 (2%)
Gram negative bacillus	51 (61%)
Enterobacteria	38 (45%)
Groupe 0	
*P. mirabilis*	1 (1%)
Groupe 1	
*E. coli*	12 (14%)
Groupe 2	
*K. pneumoniae*	5 (6%)
*K. oxytoca*	3 (4%)
*C. koseri*	2 (2%)
Groupe 3	
*Enterobacter* spp.	5 (6%)
*K. aerogenes*	2 (2%)
*C. freundii*	1 (1%)
*M. morganii*	1 (1%)
*H. alvei*	1 (1%)
Groupe 5	
*Proteus vulgaris*	5 (6%)
Coccobacilli	
*Haemophilus* spp.	10 (12%)
Vibrio	
*Campylobacter* spp.	2 (2%)
Others	
*Stenotrophomonas* spp.	1 (1%)
Anaerobic	8 (9%)
Dose of cefotaxime (g/d)	6 (6–8) [4–24]
Duration between introduction and collection (h)	38.5 (24.3–65.6) [10–206]
Cefotaxime blood concentration (mg/L)	32 (21.8–47.9) [4.2–120]
<25	23 (33%)
25–60	38 (54%)
>60	9 (13%)
SOFA (/24) at cefotaxime introduction	9 (5–11) [0–18]
Mechanical Ventilation	52 (74%)
Renal Replacement Therapy	3
Length of stay in ICU (d)	19 (11–31) [1–97]
Number of death in ICU	15 (21%)

## Data Availability

The datasets used and/or analysed in the current study are available from the corresponding author upon reasonable request.
